# A regulatory circuit of lncRNA NLGN1-AS1 and Wnt signalling controls clear cell renal cell carcinoma phenotypes through FZD4-modulated pathways

**DOI:** 10.18632/aging.204263

**Published:** 2022-09-27

**Authors:** Haifeng Gao, Wei Chen, Gaojian Pan, Hui Liu, Jinke Qian, Weijun Tang, Wei Wang, Shilei Qian

**Affiliations:** 1 Department of Urology, Binhai County People’s Hospital, Yancheng 224500, China; 2 Department of Urology, Nanjing First Hospital, Nanjing Medical University, Nanjing 210006, China; 3 Department of Urology, Yancheng Third People’s Hospital, Yancheng 224000, China; 4 Department of Oncology, Huaian Hospital of Huaian City, Huai’an 223200, China

**Keywords:** clear cell renal cell carcinoma, long noncoding RNA, NLGN1-AS1, proliferation, Wnt/β-catenin pathway

## Abstract

Background: Recent evidence has indicated that long non-coding RNAs (lncRNAs) were emerged as key molecules in clear cell renal cell carcinoma (ccRCC). TCGA database showed that the expression level of lncRNA NLGN1-AS1 was up-regulated in ccRCC; However, whether NLGN1-AS1 implicated in the malignant progression of ccRCC remained unclear.

Methods: Based on TCGA database, candidate lncRNAs were selected and quantitative real-time PCR (qRT-PCR) was utilized to verify the expression levels of candidate lncRNAs in human ccRCC tissues. Loss-of-function experiments were performed to examine the biological functions of NLGN1-AS1 both *in vitro* and *in vivo*. According to bioinformatics analysis, fluorescence reporter assays and rescue experiments, the underlying mechanisms of NLGN1-AS1 in ccRCC cell lines were so clearly understood.

Results: As a novel lncRNA, NLGN1-AS1 was up-regulated in ccRCC cell lines and associated with poor prognosis of and ccRCC patients, which was correlated with the progression of ccRCC. Functionally, the down-regulation of NLGN1-AS1 significantly decreased the proliferation of ccRCC cells both *in vitro* and *in vivo*. Bioinformatics analysis and luciferase report assays identified that miR-136-5p was a direct target of NLGN1-AS1. We also determined that FZD4 were inhibitory targets of miR-136-5p, and that Wnt/β-catenin signaling was inhibited by both NLGN1-AS1 knockdown and miR-136-5p over-expression. In addition, we found that the suppression of proliferation and the inhibition of Wnt/β-catenin pathway induced by NLGN1-AS1 knockdown would require the over-expression of FZD4.

Conclusions: Our findings suggested that lncRNA NLGN1-AS1 could promote the progression of ccRCC by targeting miR-136-5p/FZD4 and Wnt/β-catenin pathway, and might serve as a novel potential therapeutic target to inhibit the progression of ccRCC.

## INTRODUCTION

Renal cell carcinoma (RCC), as the third most prevalent malignant tumor of the urinary system, accounting for 4% around of adult malignancies [1, 2]. According to pathological types, clear cell RCC (ccRCC), representing 75–80% of RCC, originates from epithelial cells in the proximal renal tubule [3]. Accumulating clinical evidence has demonstrated that ccRCC had been positive correlated with high rate of metastasis and dismal prognosis, due to resisting radiotherapy and chemotherapy [4, 5]. Therefore, nearly one-third of ccRCC patients have been found the localized or distant metastasis, when receiving surgery, and for a few patients with advanced ccRCC, anti-angiogenesis drugs that target VEGF signaling pathway would be chosen [6]. However, due to the limitation of therapeutic effects, a part of these patients might relapse [7]. The multifactorial nature and complexity of RCC pathogenesis have been identified as major barriers to improving patient survival. Thus, it is urgent to find a novel target against the proliferation and metastasis to improve the diagnosis, treatment and prognosis of ccRCC patients.

Long non-coding RNAs (lncRNAs) represent an eukaryotic cell genome-encoded transcripts, with restricted potentiality to encode proteins whose nucleotides length is over 200 [8, 9]. Functionally, the rapidly growing evidence has shown that lncRNAs could regulate gene expression in several cellular processes, including cell proliferation, migration, invasion and apoptosis [10, 11]. Mechanistically, some researches imply that lncRNAs may be involved in enlisting chromosome remodeling complexes in gene promoters [12, 13]. In addition, lncRNAs play an important role in functioning as sponges to block miRNA [14–16]. Furthermore, lncRNAs can interact directly with a number of key proteins to increase or decrease their expression [17, 18]. Thus, abnormal expression or functional changes of lncRNAs are closely related to the progression of genitourinary malignancies, which have been revealed in a number of studies [19–21]. However, the mechanism by which lncRNAs play a vital part in ccRCC disease, would be uncertain.

This study is the first to pay attention to expression pattern, clinical characteristics and potential molecular mechanisms of lncRNA NLGN1-AS1 in the development of ccRCC. Quantitative real-time PCR (qRT-PCR) assay identified that NLGN1-AS1 expression level was significantly up-regulated in ccRCC cell lines. Interestingly, our findings suggested that the lncRNA NLGN1-AS1 accelerated ccRCC proliferation by activating Wnt/β-catenin pathway and promoted Frizzled receptor-FZD4 expression, providing ccRCC with a novel potential treatment regimen.

## MATERIALS AND METHODS

### TCGA analysis

According to the Cancer Genome Atlas (TCGA) Data Portal (https://tcga-data.nci.nih.gov/tcga/), the RNA-seq data of ccRCC samples and matched normal samples were downloaded. Date quantification for the expression levels of lncRNAs was performed by a series of steps that quality analysis, alignment, and expression quantification.

### RCC patients and tissue specimens

All twenty ccRCC samples and adjacent healthy tissue samples were attained from ccRCC patients after nephrectomy in Urological Department, Binhai County People’s Hospital (Yancheng, China). No patients with ccRCC were given preoperative chemotherapy or radiotherapy. Fresh tissue samples after resection from ccRCC patients were immediately frozen in liquid nitrogen to prevent the protein or RNA from breaking down.

### RCC cell lines and culture conditions

The human ccRCC cell lines (786-O, 769-P, CaKi-1, CaKi-2, ACHN, A498 and OSRC-2), human renal tubular epithelial cells (HK-2) and human embryonic kidney (HEK) 293FT cell line were all bought from Cell Bank of the Chinese Academy of Sciences (Shanghai, China). 786-O, 769-P and OSRC-2 cells were cultured in RMPI 1640 (Gibco, USA). CaKi-1 and CaKi-2 cells were cultured in McCoy’s 5A Medium (Gibco, USA). ACHN and A498 cells were cultured in Dulbecco’s Modified Eagle’s Medium (DMEM, Gibco, USA). HEK-293FT cells were cultured in Minimum Essential Medium (MEM, Gibco, USA). HK-2 cells were cultured in Kaighn’s Modification of Ham’s F-12 Medium (KM, ScienCell, USA). All medium was added with 10% Fetal Bovine Serum (FBS, Hyclone, USA) with 1% Penicillin/Streptomycin (P/S, Gibco, USA). All cells were kept in the humidified cell incubator (37°C, 5% CO_2_).

### Cell transfection and lentivirus production

In accordance with the manufacturer’s instructions, ccRCC cells were transfected with oligonucleotides and plasmids using Lipofectamine 3000 (Invitrogen). The cells were selected with Puromycin (2 μg/mL) for 2 weeks to establish ccRCC cell lines with stable NLGN1-AS1 knockdown or FZD4 over-expression after 48 h transfection; and short hairpin RNA (shRNA) was also transfected into ccRCC cells, too. QRT-PCR was used to verify transfection efficiency. The lentivirus-containing shRNA targeting NLGN1-AS1 and the pLenti-GIII-CMV vector for FZD4 over-expression were bought from Servicebio (Wuhan, China), and has-miR-136-5p mimic, and negative control (NC) oligonucleotides were bought from Realgene (Nanjing, China).

### Cell proliferation assay

We transfected 769-P and Caki-1 cells to investigate the transfection effect on the proliferative capacity by using cell proliferation assay. Different pre-treated cells were seeded into 96-well plates at a density of 3000 cells/well and successively cultured for 8, 24, 48, and 72 h. Based on the product instructions, each well was added with 20 μL Cell Counting Kit-8 (CCK-8; Dojindo Molecular Technologies, Japan) daily to calculate the number of cell proliferation. The absorbance of the solution was measured at 450 nm. All data were represented as mean ± SD of three independent wells in each group.

### Colony formation assay

769-P and Caki-1 cells (0.2 × 10^3^ cells per well) were seeded in a six-well plate and cultured for 14 days after transfection. Afterwards, these cells were fixed with 4% paraformaldehyde for 20 min and stained for 20 min with 0.5% crystal violet. Finally, the colonies were counted using Image J, and images were taken using Quantity One software (Bio-Rad, Hercules, CA, USA). All data were represented as mean ± SD of three independent experiments.

### RNA extraction and quantitative reverse transcription PCR (qRT-PCR)

The total RNA was extracted from frozen ccRCC tissues and cultured cells though TRIzol reagent (Invitrogen, Carlsbad, CA, USA). One microgram of total RNA was reverse-transcribed to high-quality cDNA using a PrimeScript RT Master Mix (Vazyme Biotech, Nanjing, China). qRT-PCR was proceeded with the SYBR Green Mix (Vazyme Biotech, Nanjing, China). The expression level of miRNAs was detected by All-in-One^™^ miRNA qRT-PCR Detection Kit (Vazyme Biotech, Nanjing, China). All qRT-PCR assays were proceeded with an ABI 7500 system (Applied Biosystems, Foster City, CA, USA). The housekeeping gene glyceraldehyde 3-phosphate dehydrogenase (GAPDH) or SNORD6 (U6 snRNA) was chosen to normalize the expression levels of gene or miRNA. Data were analyzed using the 2−ΔΔCt method by Applied Biosystems StepOne Plus Real-Time PCR System (Applied Biosystems, USA). All data were expressed as mean ± SD of three independent experiments. The primer sequences (Realgene, Nanjing, China) were shown below.

NLGN1-AS1: Forward: 5′-GGGGGAGGGGATACTT CTGT-3′, Reverse: 5′-TGGTTGCTCTGTGCTTCCTT-3′; FZD4: Forward: 5′-AACGTGACCAAGATGCCC AA-3′, Reverse: 5′-TGATCAACTTGGCATGGGCT-3′; GAPDH: Forward: 5′-TGACTTCAACAGCGACA CCCA-3′, Reverse: 5′-CACCCTGTTGCTGTAGCC AAA-3′; miR-136-5p: Forward: 5′-ACACTCCAGCT GGGACTCCATTTGTTTT-3′, Reverse: 5′-CCAGTG CAGGGTCCGAGGT-3′; U6: Forward: 5′-CTCGCTTC GGCAGCACA-3′, Reverse: 5′-AACGCTTCACG AATTTGCGT-3′.

### Fluorescence *in situ* hybridization (FISH)

RNA FISH assay was utilized for locating NLGN1-AS1, while Olympus confocal laser scanning microscope was utilized for acquiring images. Paraffin-embedded tissue blocks were retrospectively retrieved from ccRCC patients. Quantum dot fluorescence *in situ* hybridisation (QD-FISH) utilizes digoxigenin-labelled oligonucleotide probes to indirectly label digoxigenin antibody-bound quantum dots to locate NLG1-AS1.

### Immunohistochemistry (IHC)

Hematoxylin and eosin (H&E) staining was applied for choosing representative areas. IHC for the target molecules was performed on paraffin sections using a primary antibody against FZD4 and Ki67, and the proteins *in situ* were visualized with 3, 3-diaminobenzidine. All antibodies obtained from Servicebio (Wuhan, China). Regarding histological scoring, three randomly selected high magnification fields (×400) from tissues and normal kidney tissues were assessed independently by a minimum of two experienced pathologists who were unaware of the pathological features of ccRCC.

### Western blotting

Total proteins from 769-P and Caki-1 cells were prepared with RIPA buffer containing protease inhibitors and phosphatase inhibitors. The lysates were centrifuged on ice for 30 min and then centrifuged at 12,000 rpm for 15 min at 4°C. The protein concentration was detected using a BCA kit (Beyotime Biotechnology, Beijing, China). Proteins of equal amounts (30 μg) were electrophoresed through an 8–10% sodium dodecyl sulfate/polyacrylamide gel and transferred onto nitrocellulose membranes (Millipore, Billerica, MA, USA). After blocking antigen, the membranes were hybridized with an appropriate primary antibody at 4°C overnight. The membranes were then incubated with the corresponding secondary antibody at room temperature for 1 h. The expression of GAPDH was used as loading control. Based on instructions, protein bands were observed using a chemiluminescence reagent (ECL) kit (Beyotime Biotechnology). The information of antibodies was listed as follow: Wnt3, GSK3β, FZD4, β-catenin, GAPDH (Servicebio, Wuhan, China).

### Ethynyl-2′-deoxyuridine (EDU) assay

In accordance with the manual, the proliferative capacity of ccRCC cell lines was measured by EDU kit (Abcam, Cambridge, MA, USA). These ccRCC cells, which grow on cover slips, were cultured with EDU during DNA synthesis for 2 h, and were stained with anti-EDU antibody after treatment. Images were obtained by Olympus camera under a microscope.

### Luciferase reporter assay

NLGN1-AS1 wild type with potential binding site of miR-136-5p or mutant of each sites was fused to the pmirGLO Dual-Luciferase miRNA Target Expression Vector (Realgene, Nanjing, China). The reporter vectors named pmirGLO-FZD4-WT and pmirGLO-FZD4-Mut were formed as above. HEK-293FT cells were co-transfected with above vectors using Lipofectamine 3000 (Invitrogen, USA). After 48 h transfection, the luciferase activities were detected by Dual-Luciferase Reporter Assay System (Promega, Madison, WI, USA).

### Xenograft subcutaneous implantation

Female BALB/c nude mice with 4 weeks old were purchased from Model Animal Research Center of Nanjing University (Nanjing, China), and kept in Animal Core Facility of Nanjing Medical University for adapting to the environment. For the *in vivo* tumor formation assay, the ccRCC cell lines (1 × 10^7^ cells in 0.1 ml of Matrigel) were stably transfected with NLGN1-AS1/NC lentiviral vector and then injected subcutaneously into the armpit of female nude mice, respectively (*n* = 5 mice/group). The tumor volume and weight were measured every 4 days. The mice were killed on the 28^th^ days after injection. The animal studies were approved by the Nanjing Medical University’s Animal Ethics Committee.

### Statistical analysis

The data are expressed as the mean ± standard error (SD) from at least three independent experiments. The data were analyzed with SPSS 22.0 software (SPSS, Chicago, IL, USA). Differences between the two groups were tested by Student’s *t*-test or by one-way ANOVA when there are two more groups. The correlation between gene expression levels and clinicopathological variables was calculated by the chi-square test or Fisher’s exact test. *P* < 0.05 means statistically significant.

## RESULTS

### NLGN1-AS1 is a novel lncRNA involved in renal malignant transformation

**Figure 1:**
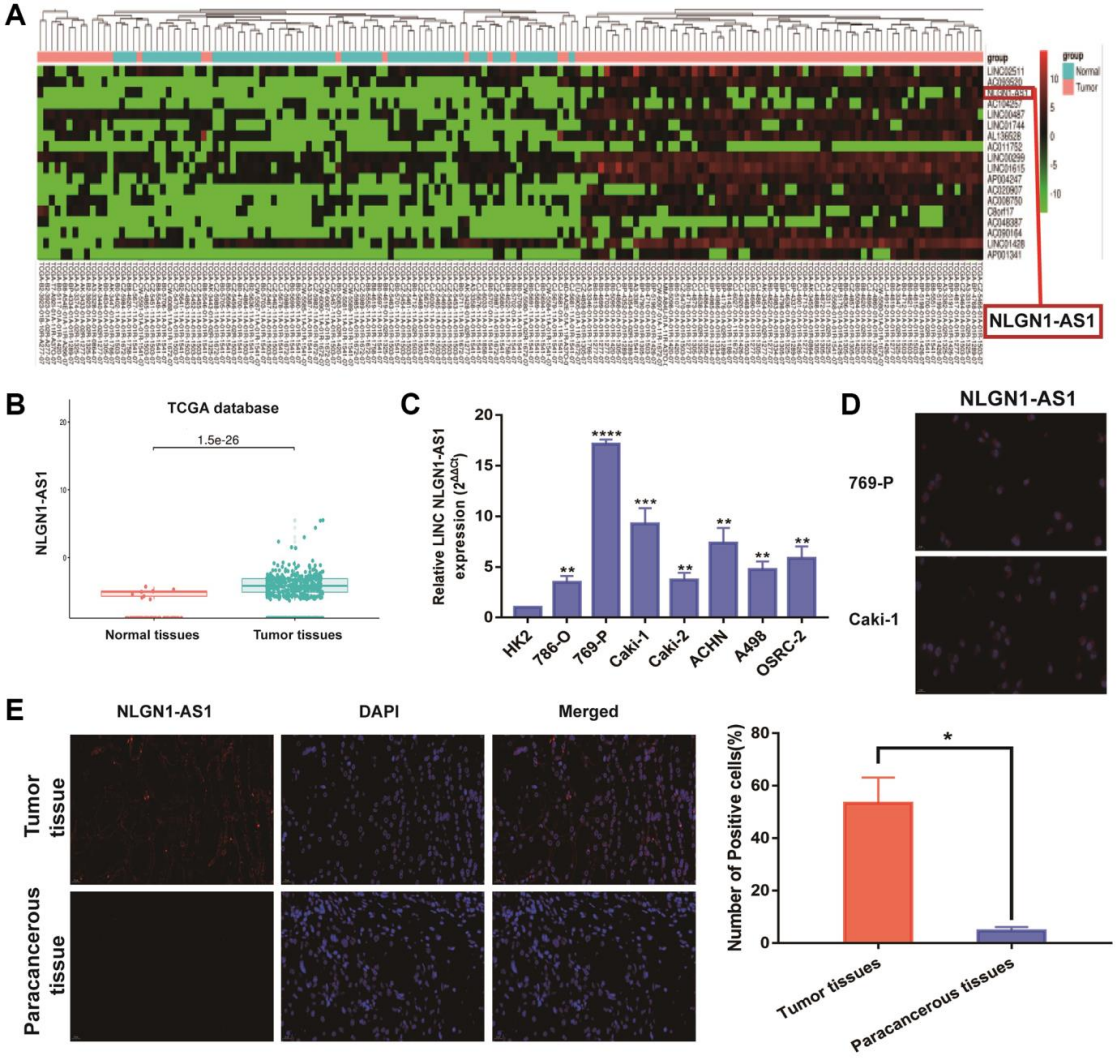
**NLGN1-AS1 is a novel lncRNA involved in renal malignant transformation.** (**A**) The heatmap of 18 lncRNA expression profiles. (**B**) NLGN1-AS1 expression in ccRCC tissues and normal tissues from TCGA KIRC dataset. (**C**) NLGN1-AS1 expression in a series of RCC cell lines (786-O, 769-P, Caki-1, Caki-2, ACHN, A498 and OSRC-2) and human normal renal tubular epithelial cell line HK-2. (**D**) Representative images of fluorescence *in situ* hybridization (FISH) assays of the subcellular localization and expression of NLGN1-AS1 (red) while the nucleuses were stained with DAPI (blue) in 769-P and Caki-1 cells. (**E**) FISH analysis of NLGN1-AS1 (red) in adjacent tissues and ccRCC tissues while the nucleuses were stained with DAPI (blue). The data represent the mean ± SD of 3 replicates. ^*^P < 0.05; ^**^P < 0.01, ^***^P < 0.001, ^****^P < 0.001.

TCGA databases were used for investigating the potential relevant lncRNAs associated with ccRCC progression. We firstly focused on candidate lncRNAs form TCGA database (Figure 1A, *P* < 0.001). As shown in Figure 1B, the expression of NLGN1-AS1 significantly up-regulated in ccRCC tissues, based on TCGA database (*P* < 0.001). Meanwhile, NLGN1-AS1 expression level was significantly higher in seven ccRCC cell lines than that in human normal renal tubular epithelial cells (HK-2 cells) by qRT-PCR analysis (Figure 1C). Moreover, it could be seen that NLGN1-AS1 (red) was found primarily in the cytoplasm, whereas the nucleuses were stained with DAPI (blue) in 769-P and Caki-1 cells (Figure 1D). Meanwhile, same experiment indicated that the high expression of NLGN1-AS1 in ccRCC tissues in comparison to adjacent tissues (Figure 1E). In brief, the data above indicated that NLGN1-AS1 might play a part in the pathogenesis of ccRCC.

### Reduced expression of NLGN1-AS1 significantly suppressed the proliferation of ccRCC cells *in vitro* and *in vivo*

**Figure 2:**
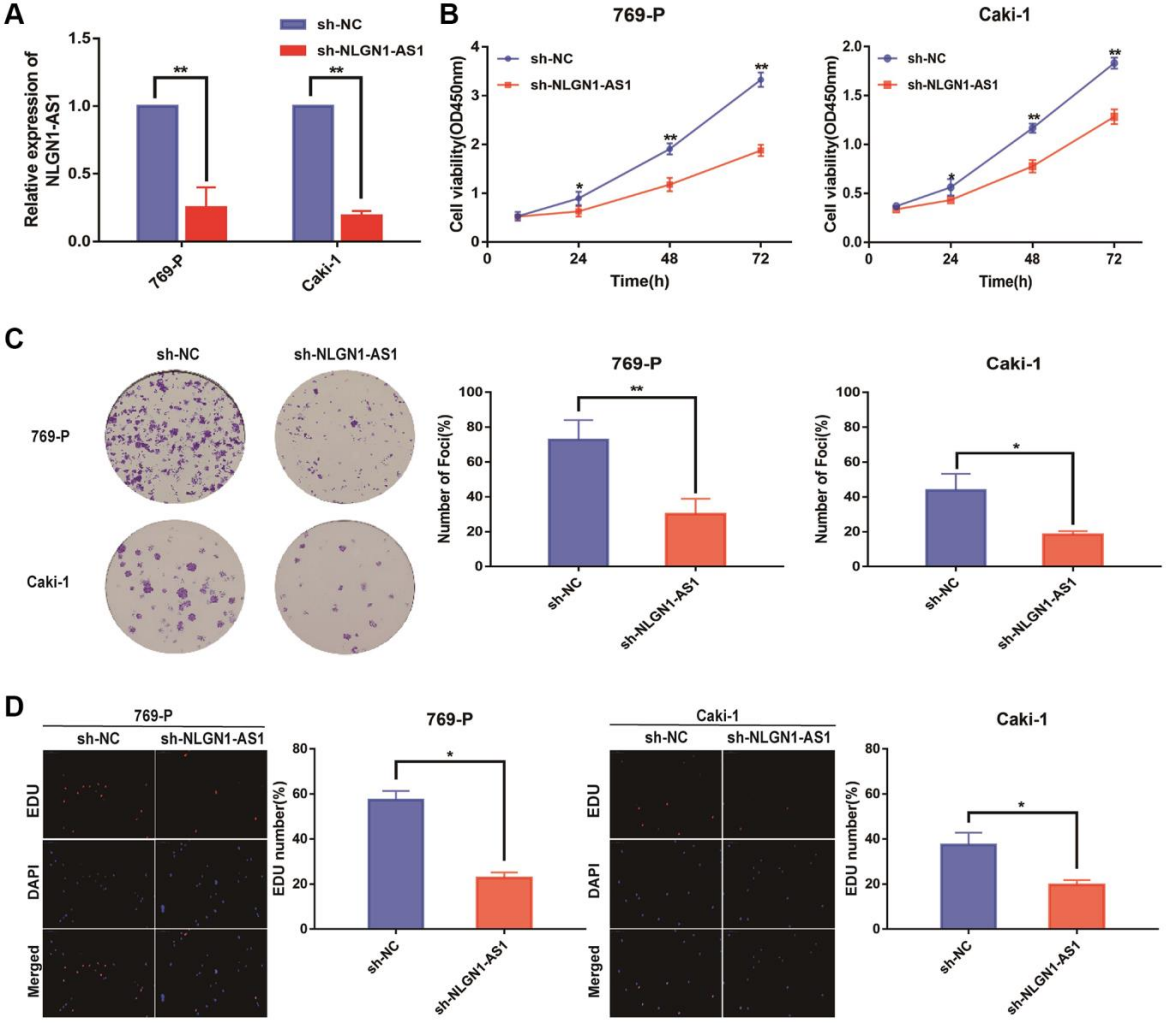
**Reduced expression of NLGN1-AS1 markedly suppressed the proliferation of ccRCC cells *in vitro*.** (**A**) qRT-PCR was conducted to verify the relative expression of NLGN1-AS1 in 769-P and Caki-1 cells transfected with independent shRNA targeting NLGN1-AS1. (**B**) CCK-8 assay performed with 769-P and Caki-1 cells after transfection of sh-NLGN1-AS1 compared with sh-NC. (**C**) Representative results of the colony formation of 769-P and Caki-1 cells transfected with sh-NLGN1-AS1 or sh-NC. The colonies were counted and captured. (**D**) Representative images of EDU assay performed with the 769-P and Caki-1 cells transfected with sh-NLGN1-AS1 or sh-NC. The data represent the mean ± SD of 3 replicates. ^*^P < 0.05; ^**^P < 0.01.

Since NLGN1-AS1 was up-regulated and positively correlated with ccRCC progression, loss-of-function experiments was utilized to ascertain if NLGN1-AS1 could impact the proliferation of ccRCC cells. First, we verified the shRNA interference efficiency of NLGN1-AS1 by qRT-PCR analysis in 769-P and Caki-1 cells (Figure 2A). CCK-8 assay was subsequently performed in 769-P and Caki-1 cells transfected with sh-NLGN1-AS1 or sh-NC, implying that knockdown of NLGN1-AS1 inhibited the growth of these cells, compared with control cells (Figure 2B). Similarly, NLGN1-AS1 knockdown significantly impaired the proliferation capacities of ccRCC cells through clone formation assay and EDU assay (Figure 2C, 2D). The entire *in vitro* experiment suggested that NLGN1-AS1 promoted ccRCC cells proliferation. The effect of NLGN1-AS1 on proliferation *in vivo* will be investigated in the future.

**Figure 3:**
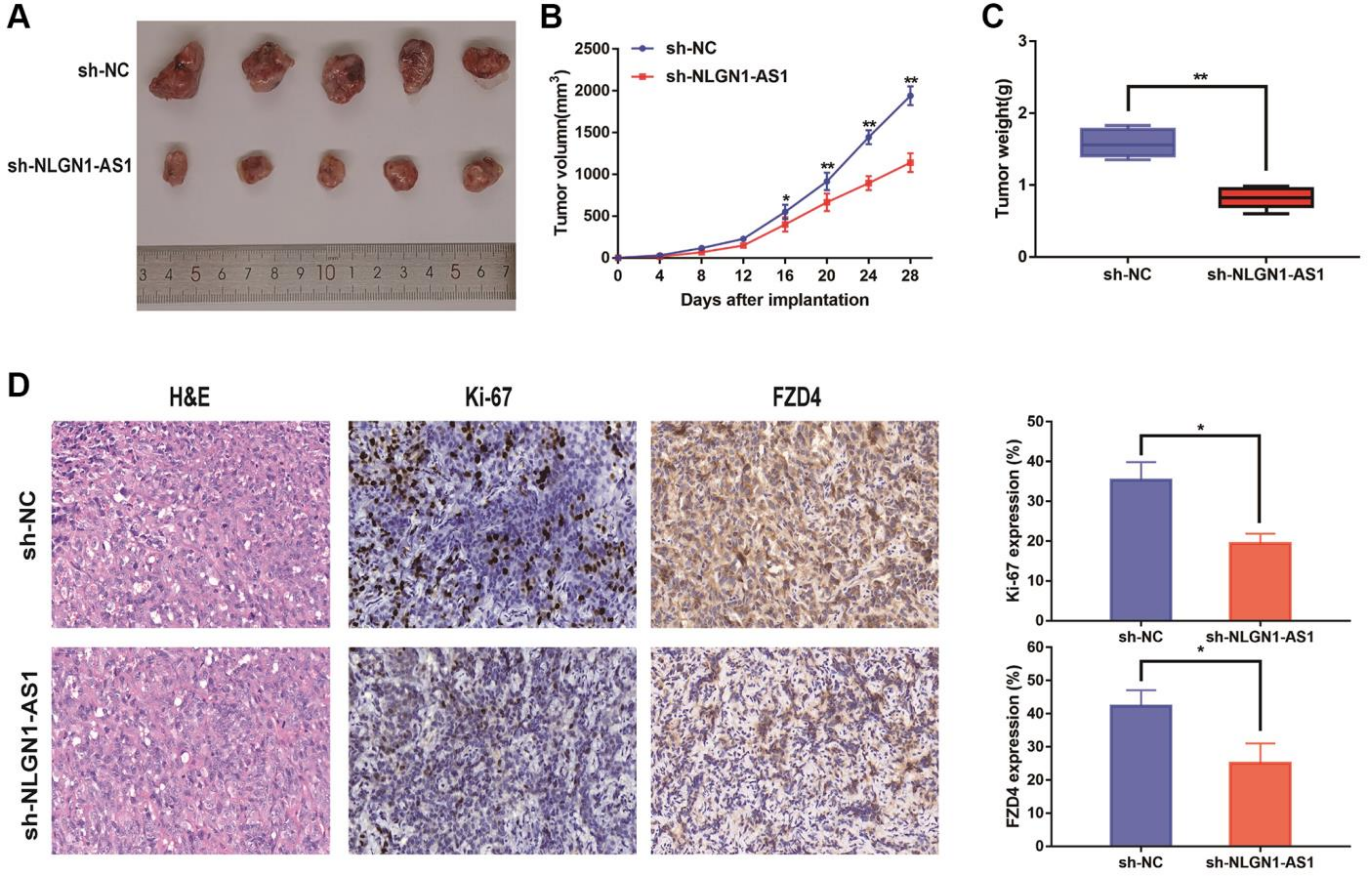
**Reduced expression of NLGN1-AS1 markedly suppressed the proliferation of ccRCC cells *in vivo*.** (**A**) The *in vivo* effect of NLGN1-AS1 was evaluated in xenograft mouse models bearing tumors originating from 769-P cells stably transfected with sh-NLGN1-AS1 or sh-NC, *n* = 5/group; Tumor volume and weight of the xenograft were shown in the right. (**B**) Tumor volume of the xenograft in each group. (**C**) Tumor weight of the xenograft in each group. (**D**) The tumor sections from sh-NLGN1-AS1 and sh-NC group of xenograft mouse models were subjected to H&E staining and immunohistochemistry staining using antibodies against ki-67 and PDZ4 (400×). The data represent the mean ± SD of 3 replicates. ^*^P < 0.05; ^**^P < 0.01.

The 769-P cells with sh-NLGN1-AS1 and their corresponding control group cells were subcutaneously injected into the right armpit of female nude mice. Expectedly, sh-NLGN1-AS1 cell-derived tumors were significantly smaller compared with the tumors created by NC group. Concurrently, the sh-NLGN1-AS1 group had significantly lighter tumor weight compared with NC group (Figure 3A–3C). The IHC figures showed that the tumor tissue of sh-NLGN1-AS1 group exhibited decreased ki-67 and FZD4 staining than those of NC group (Figure 3D). Together, we might assume that NLGN1-AS functioned as a novel tumor enhancer *in vitro* and *in vivo* by promoting tumor proliferation.

### NLGN1-AS1 bound to miR-136-5p and repressed its expression

**Figure 4:**
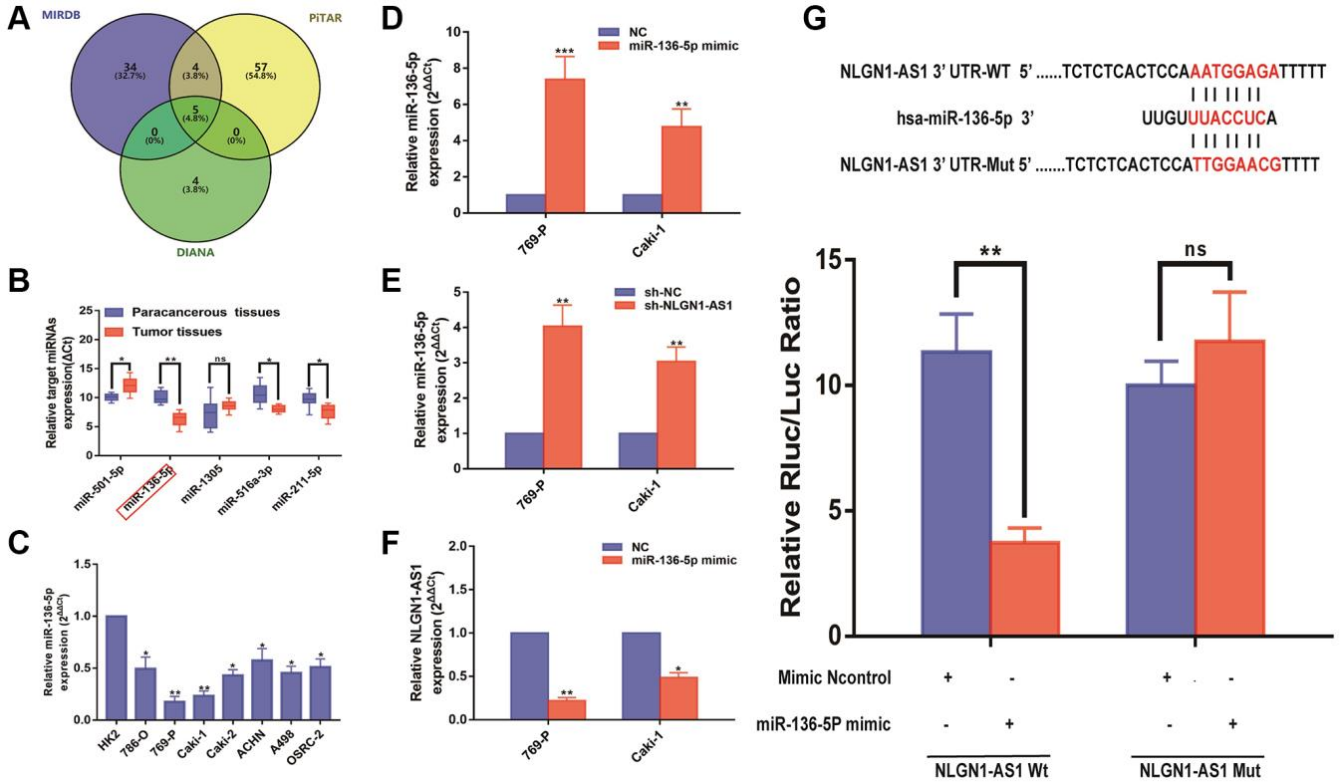
**NLGN1-AS1 binds to miR-136-5p and represses its expression.** (**A**) A schematic diagram used to search the target miRNAs of NLGN1-AS1 in three databases. (**B**) qRT-PCR assay confirmed the relative expression of five candidate miRNAs of NLGN1-AS1 in 769-P and Caki-1 cells and 20 paired ccRCC cancer tissues compared with corresponding adjacent normal tissues. (**C**) Relative expression of miR-136-5p in a series of RCC cell lines (786-O, 769-P, Caki-1, Caki-2, ACHN, A498 and OSRC-2) and human normal renal tubular epithelial cell line HK-2. (**D**, **E**) Relative expression of miR-136-5p in 769-P and Caki-1 cells transfected with miR-136-5p mimic(d) or cells after transfection with sh-NLGN1-AS1(e). (**F**) Relative expression of NLGN1-AS1 in 769-P and Caki-1 cells transfected with miR-136-5p mimic. (**G**) Schematic illustration of the predicted binding sites between NLGN1-AS1 and miR-136-5p, and mutation of potential miR-136-5p binding sequence in NLGN1-AS1. Relative luciferase activities of wild type (WT) and mutated (Mut) NLGN1-AS1 reporter plasmid in human embryonic kidney (HEK) 293T cells co-transfected with miR-136-5p mimic. The data represent the mean ± SD of 3 replicates. ^*^P < 0.05; ^**^P < 0.01.

As previous findings in Figure 1E, NLGN1-AS1 transcript was predominately localized in the cytoplasm. And competitive endogenous RNAs (ceRNAs), which sponged various miRNAs to suppress the regulatory effects on target mRNAs, was the most well-known mechanism of cytoplasmic lncRNAs [20]. In order to profoundly investigate the underlying molecular mechanism of NLGN1-AS1 regulation of ccRCC, we predicted three potential conjugated miRNAs of NLGN1-AS1 with Mirdb (http://www.mirdb.org/), PITAR (https://pictar.mdc-berlin.de/), and DIANA (http://diana.imis.athena-innovation.gr/) (Figure 4A). Among the five related miRNAs, miR-136-5p, miR-516a-3p and miR-211-5p were significantly decreased verified by qRT-PCR analysis in 20 ccRCC tissues compared with pair-normal tissues, and miR-136-5p was the largest statistical difference among them. Moreover, we further validated that miR-136-5p expression level was significantly decreased in 769-P and Caki-1 cells (Figure 4B). Furthermore, we used qRT-PCR assay to evaluate the expression level of miR-136-5p in ccRCC cell lines (Figure 4C). And, to further examine whether miR-136-5p was indeed a ceRNA for NLGN1-AS1. First, we verified the over-expression efficiency of the miR-136-5p mimic by qRT-PCR analysis in 769-P and Caki-1 cells (Figure 4D). Then, we knocked down NLGN1-AS1, and found the expression level of miR-136-5p was significant up-regulated, whereas miR-136-5p over-expression also resulted in the down-regulation of NLGN1-AS1 (Figure 4E, 4F). Hence, there might be negative feedback regulation between NLGN1-AS1 and miR-136-5p.

Then, we subcloned wild-type (NLGN1-AS1-Wt) and mutated (NLGN1-AS1-Mut) miR-136-5p binding site into dual-luciferase reporters. Consistently, in the group of NLGN1-AS1-Wt, the luciferase activity was obviously inhibited in the miR-136-5p mimic group in comparison to control group. Nevertheless, no significant changes above were seen in mutated NLGN1-AS1 group, indicating that NLGN1-AS1 might be the target binding site of miR-136-5p (Figure 4G).

### NLGN1-AS1 was associated with the activity of the Wnt/β-catenin pathway and increased the expression level of FZD4

**Figure 5:**
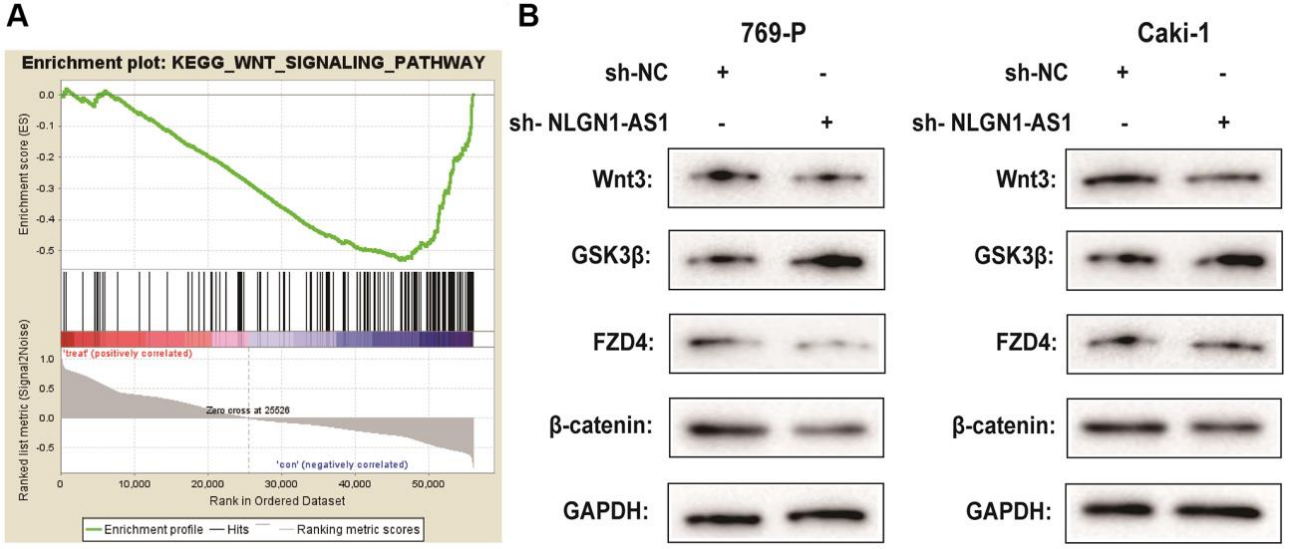
**NLGN1-AS1 is associated with the activity of the Wnt/β-catenin pathway and suppressed the expression of FZD4.** (**A**) Kyoto Encyclopedia of Genes and Genomes analysis revealed that the Wnt signaling pathway was significantly altered in ccRCC. (**B**) The effect of NLGN1-AS1 on the Wnt/β-catenin signaling pathway was analyzed by western blotting with the indicated antibodies and samples from the 769-P and Caki-1 cells transfected with sh-NLGN1-AS1 or sh-NC. The data represent the mean ± SD of 3 replicates. ^*^P < 0.05; ^**^P < 0.01.

It is confirmed that lncRNAs is greatly involved in the regulation of ccRCC progression via Wnt/β-catenin signaling pathway [21, 22]. To determine the potential mechanisms by which NLGN1-AS1 acts on ccRCC, we integrated mRNAs that were the potential related genes of NLGN1-AS1 by bioinformatical analysis, which also contained FZD4 gene. Furthermore, we investigated the signaling pathways that were potentially related mRNAs involved in using Kyoto Encyclopedia of Genes and Genomes analysis. Notably, a significant alteration of Wnt/β-catenin pathway in ccRCC was observed in accordance with *in vitro* the findings (Figure 5A). To test the effect of NLGN1-AS1 over-expression on the Wnt/β-catenin signaling pathway, the result by Western blotting assay suggested that sh-NLGN1-AS1 could blunt Wnt/β-catenin signaling pathway in both 769-P and Caki-1 cells. Compared with NC group, the NLGN1-AS1 knockdown group exhibited the decrease of the expression of Wnt3, FZD4 and β-catenin, conversely, the total level of GSK3β was also increased in both 769-P and Caki-1 cells transfected with sh-NLGN1-AS1 (Figure 5B).

### FZD4 is a direct target of miR-136-5p in ccRCC progression

**Figure 6:**
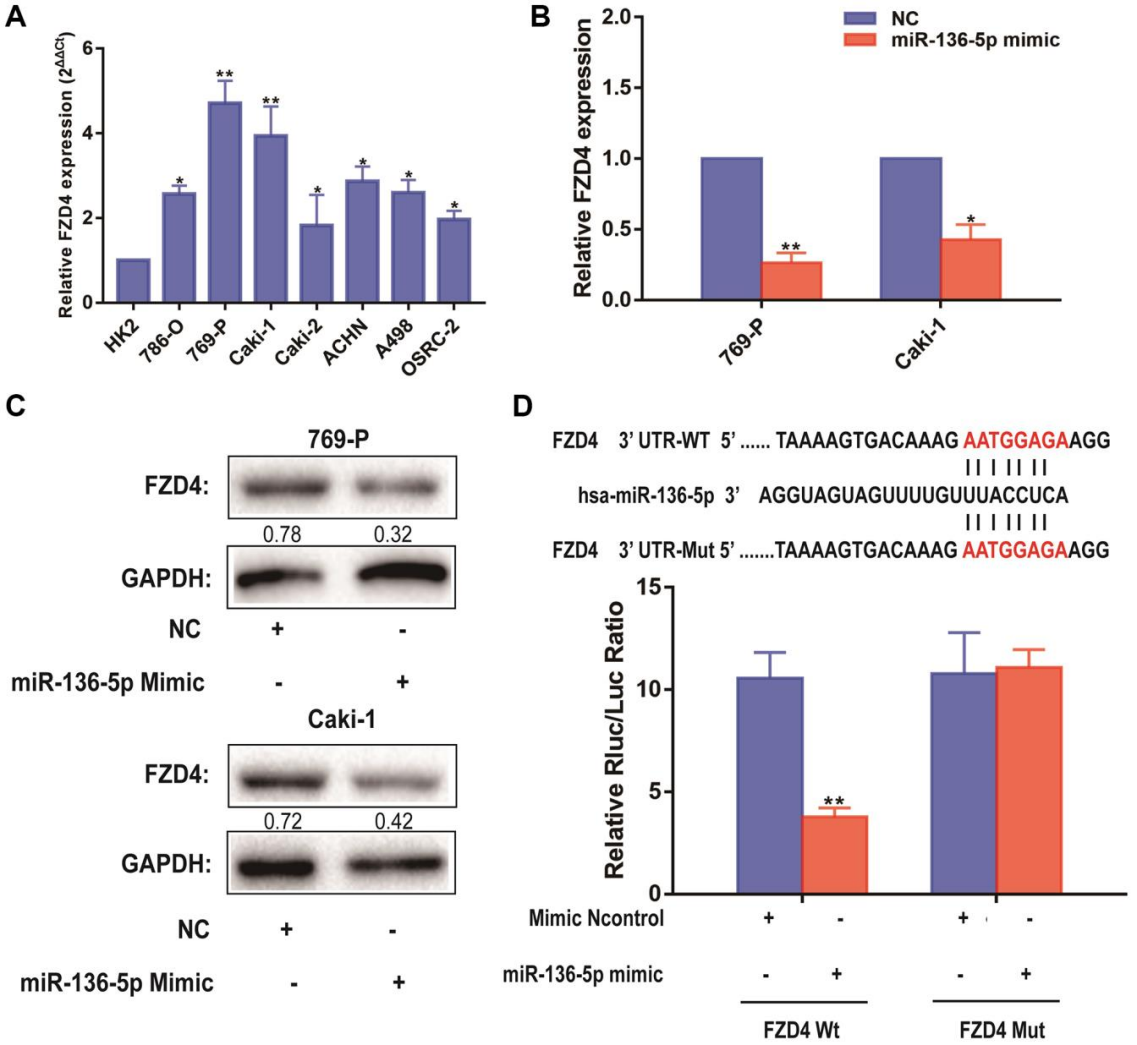
**FZD4 is a direct target of miR-136-5p in ccRCC progression.** (**A**) Relative expression of FZD4 in a series of RCC cell lines (786-O, 769-P, Caki-1, Caki-2, ACHN, A498 and OSRC-2) and human normal renal tubular epithelial cell line HK-2. (**B**) Relative expression of FZD4 in 769-P and Caki-1 cells transfected with miR-136-5p mimic. (**C**) Western blots analysis of FZD4 in miR-136-5p mimic 769-P and Caki-1 cells compared with NC group, GAPDH was used as a loading control. (**D**) Schematic illustration of the predicted binding sites between FZD4 and miR-136-5p, and mutation of potential miR-136-5p binding sequence in FZD4. Relative luciferase activities of wild type (WT) and mutated (Mut) FZD4 reporter plasmid in human embryonic kidney (HEK) 293T cells co-transfected with miR-136-5p mimic. The data represent the mean ± SD of 3 replicates. ^*^P < 0.05; ^**^P < 0.01.

Besides, it can be seen that the expression level of FZD4, which was related to Wnt/β-catenin pathway, was significantly higher in ccRCC cell lines than that in HK-2 cells, which revealed by qRT-PCR analysis (Figure 6A). MiRNAs play a variety of biological functions primarily through inhibition of mRNA translation or degradation of mRNA [23]. According to previous studies above, we focused on the FZD4 gene, a Wnt/β-catenin pathway related mRNA, as the primary candidate. To further uncover the relevance between NLGN1-AS1 and miR-136-5p, we firstly validated that FZD4 expression was significantly decreased in 769-P and Caki-1 cells transfected with miR-136-5p mimic (Figure 6B). Furthermore, the protein expression of FZD4 was measured in 769-P and Caki-1 cells transfected with miR-136-5p mimic or NC mimic (Figure 6C). And these results as we expected, compared with NC group, FZD4 expression was decreased in miR-136-5p mimic group. We then performed luciferase reporter assay by co-transfecting luciferase reporter plasmids with miR-136-5p (Figure 6D). The over-expression of miR-136-5p decreased the luciferase activity driven by FZD4-WT in HEK293FT cells, whereas it did not alter FZD4-Mut activity, indicating that FZD4 was the direct target of miR-136-5p.

### NLGN1-AS1 attenuates the proliferation abilities of ccRCC cells through FZD4/Wnt signaling pathway

**Figure 7:**
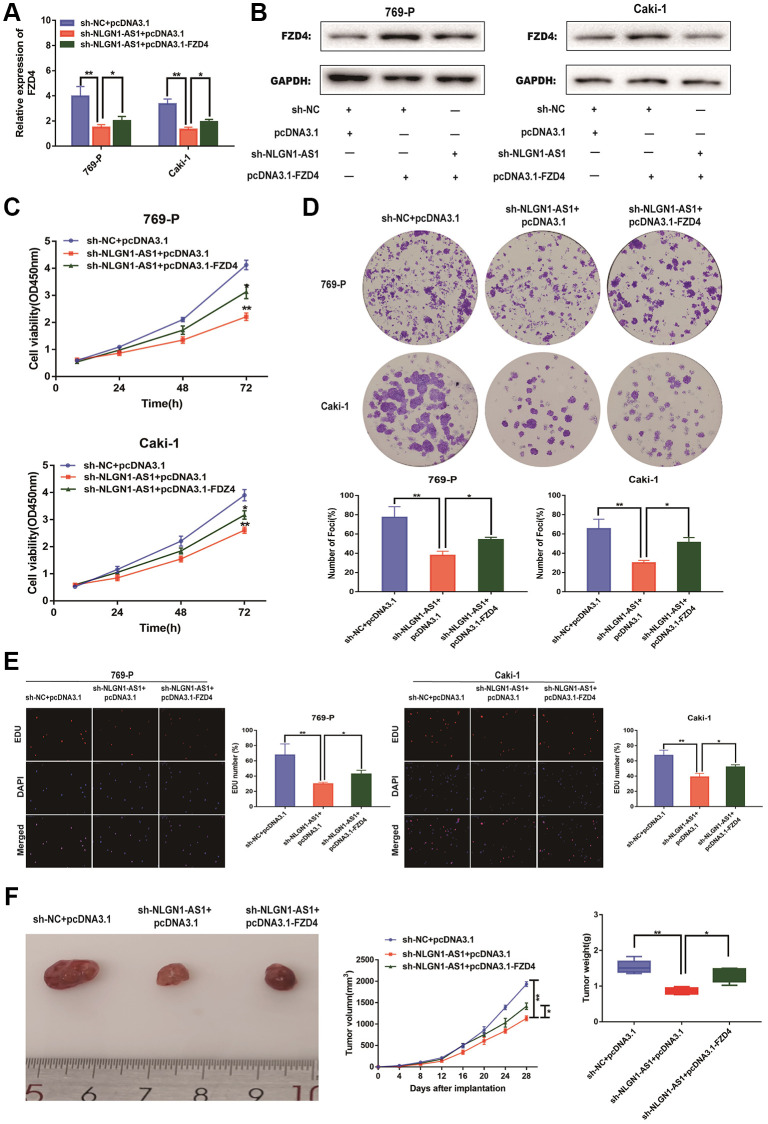
**NLGN1-AS1 attenuates the proliferation abilities of ccRCC cells through FZD4/Wnt signaling pathway.** (**A**) qRT-PCR was conducted to verify the relative expression of FZD4 in 769-P and Caki-1 cells transfected with sh-NLGN1-AS1 or oe-FZD4. (**B**) The expression of FZD4 was analyzed by western blotting with the indicated antibodies and samples from the 769-P and Caki-1 cells transfected with sh-NLGN1-AS1 or oe-FZD4. (**C**–**E**) CCK-8 assay, colony formation analysis, and EDU assay in 769-P and Caki-1 cells transfected with sh-NC+oe-NC, sh-NC+oe-FZD4 and sh-NLGN1-AS1+oe-FZD4. (**F**) *In vivo* evaluating in xenograft mouse models bearing tumors originating from 769-P cells stably transfected with sh-NC+oe-NC, sh-NC+oe-FZD4 and sh-NLGN1-AS1+oe-FZD4, *n* = 5/group; Tumor volume and weight of the xenograft were shown in the right. The data represent the mean ± SD of 3 replicates. ^*^P < 0.05; ^**^P < 0.01.

Our previous results demonstrated that NLGN1-AS1 knockdown resulted in the suppression of ccRCC proliferation. The over-expression of NLGN1-AS1 inhibited the expression level of miR-136-5p, and miR-136-5p mimic inhibited the expression level of FZD4, in turn influencing Wnt/β-catenin signaling pathway. Consequently, the suppression of ccRCC cell proliferation resulted from NLGN1-AS1 knockdown might be caused by a reduced expression of FZD4, which ultimately inhibited the Wnt/β-catenin signaling pathway. To test this hypothesis, we first concomitantly over-expressed FZD4 in NLGN1-AS1 knockdown ccRCC cells, we first verified the expression efficiency of FZD4 by qRT-PCR analysis in 769-P and Caki-1 cells of different transfected groups (Figure 7A). The result of Western blotting consistently indicated that simultaneous knockdown of NLGN1-AS1 was able to reverse the repression of FZD4 (Figure 7B). In addition, we found that the phenotype induced by sh-NLGN1-AS1 could be partially saved by excessive expression of FZD4 using CCK-8 assay in 769-P and Caki-1 cells (Figure 7C). Similarly, the anti-proliferation activity of NLGN1-AS1 knockdown was partly reversed by co-transfection with FZD4 over-expression through clone formation assay and EDU assay in 769-P and Caki-1 cells (Figure 7D, 7E). To explore the roles of NLGN1-AS1 and FZD4 in tumor growth *in vivo*, NLGN1-AS1 knockout resulted in a lower incidence of tumor and a significant reduction in tumor size compared with sh-NC+pcDNA3.1, whereas the results showed that FZD4 over-expression had a reversal of the effect of NLGN1-AS1 on tumor growth. (Figure 7F). Altogether, these data showed that NLGN1-AS1 could enhance proliferative capacity of ccRCC through FZD4/Wnt/β-catenin signaling pathway.

## DISCUSSION

Lately, lncRNAs have been found to express abnormally in a variety of human cancers, and increasing evidence suggests that lncRNAs contribute to carcinogenesis [24–26]. Numerous research have found dysregulation of lncRNAs participated in renal carcinoma including ccRCC, suggesting that lncRNAs might serve as a new biomarker for tumors prognosis and treatment [27–31]. The lncRNA NLGN-AS1 was oriented in an antisense direction to the protein-coding gene NLGN-AS1 on the opposite DNA strand. There is no evidence that NLGN-AS1 expression was abnormal in the carcinogenesis tissue of patients, the mechanism of NLGN-AS1 in tumors and other diseases, especially ccRCC, was also unclear. Therefore, this study focused on the regulation of ccRCC progression by a new identified lncRNA NLGN-AS1 through a combined bioinformatics and experiments. We identified that the transcriptional level of NLGN-AS1 was substantially up-regulated in ccRCC tissue in comparison to surrounding normal tissues, similarly in ccRCC cell lines, and this result was also corroborated by the analysis of available data based on the TCGA database. In addition, the higher expression level of NLGN-AS1 was associated with tumor stage and positively correlated with poor prognosis of ccRCC patients. We also confirmed that NLGN-AS1 blocked tumor growth both *in vitro* and *in vivo*, which indicated that NLGN-AS1 might play an essential part in the occurrence and progression of ccRCC. Consequently, NLGN-AS1 could serve as a potential diagnostic and prognostic biomarker or even a therapeutic target for improving ccRCC patients’ outcome.

LncRNAs, which are thought to be RNA polymerase II by-products, have been shown to play a vital role in regulating a variety of physiological and pathological processes [32, 33]. The function of lncRNAs have been verified in the proliferation, metastasis, energy metabolism and drug resistance of cancer [15, 34–37]. Lately, growing research have highlighted the critical role of lncRNAs in cancer proliferation. LncRNA ANRIL, a lncRNA highly expressed in pediatric medulloblastoma, interacted with miR-323 and activated Wnt signaling pathway [38]. Atianand et al. revealed that lincRNA-EPS could specifically localize to the genomic loci of immune response genes to control gene repression [39]. In general, lncRNAs can be used to activate or suppress gene expression via the recruitment of a variety of remodeling complexes to the gene promoter [40]. Growing evidence suggests that lncRNAs have distinct biological functions primarily because of their different subcellular localization [41]. Cytoplasmic lncRNAs can act as a decoy for miRNAs, which may regulate mRNA stability or translation and ultimately affect signaling pathways [20]. We identified miR-136-5p as a target of NLGN1-AS1 by bioinformatic analyses and luciferase reporter assays. Although miR-136-5p was a key regulator of the progression of human cancer [42], no studies involving the function and mechanism of miR-136-5p in ccRCC have been conducted yet. Wang et al. [43] reported that the role of circOSBPL10 in gastric cancer was as a specific molecular sponge for miR-136-5p. Similar mechanisms of ceRNA in other diseases have confirmed the findings for ccRCC. The inverse correlation between the expression levels of NLGN1-AS1 and miR-136-5p was revealed in clinical samples. And also the interplay between changes in the expression level of NLGN1-AS1 and change of miR-136-5p expression level was verified in ccRCC cell lines, suggesting that miR-136-5p acted as a ceRNA of NLGN1-AS1. The inverse correlation between the expression levels of NLGN1-AS1 and miR-136-5p in both ccRCC clinical samples and ccRCC cell lines further validated the target relation of them. To study the functional roles of NLGN1-AS1 and miR-136-5p in regulating ccRCC progression, this is helpful to improve the reliability of our results by preventing our study from being confined to a single line of 769-P and Caki-1 cells. This study also indicated that the inhibition of NLGN1-AS1 resulted in the suppression of cell proliferation. Also, the fact that over-expression of miR-136-5p induced the same phenotypes as NLGN1-AS1 knockdown suggested the inhibition of miR-136-5p by NLGN1-AS1.

Every barber knew that the most classical pathway in ccRCC proliferation was the Wnt/β-catenin signaling pathway, which was a highly conserved signaling pathway that consisted of a number of oncogene and antioncogene proteins [44, 45]. FZD4, frizzled class receptor 4, belongs to frizzled gene family. Most frizzled receptors are coupled to the beta-catenin canonical signaling pathway, thus suppressing the processes that activate oncogenic transformation, cell proliferation, and inhibition of apoptosis. Nevertheless, the function of FZD4 in ccRCC was unknown. Differential expression of FZD4 has been linked to epigenetic changes in certain cancers, including pancreatic cancer, bladder cancer, and colorectal cancer [44–46]. Moreover, we found that knockdown of NLGN1-AS1 effectively abrogated FZD4 expression and impeded cell growth. Furthermore, it has been reported that FZD4 can activate multiple signaling pathways in human tumors [47–50]. In clinical specimen, we substantiated the up-regulated NLGN1-AS1 was potent to promote FZD4 expression, and *in vitro*, we verified that the proteins (Wnt3, FZD4, and β-catenin) associated with Wnt/β-catenin signaling pathway were over-expression when NLGN1-AS1 was up-regulated. So we got a conclusion that FZD4 was the downstream gene of NLGN1-AS1, which enhanced Wnt/β-catenin signaling pathway, and then contributed to tumorigenesis of ccRCC.

Recently, several studies have discovered that miRNAs could influence cell function by degrading or inhibiting mRNAs in cancer signaling pathways [22–24]. The Wnt/β-catenin pathway has been shown to be a key factor in the development of embryos and tumor progression [47–50]. There is increasing evidence that miRNAs regulate tumor proliferation through the interaction with related mRNAs of the Wnt/β-catenin in ccRCC. [51, 52]. Yue et al. [51] demonstrated that miR-301a promoted radiation resistance by directly targeting TCEAL7 to activate Wnt/β-catenin signaling pathway. The miR-504 hindered the mesenchymal phenotype of glioblastoma through downregulating FZD7 and impacting E-cadherin/β-catenin [52]. Our study appeared to support the FZD4 gene was a direct target of miR-136-5p via target prediction. This prediction was verified by several lines of evidence. Mechanically, we further verified the direct evidence of the interaction between FZD4 and miR-136-5p by luciferase reporter assay. We found that the wild type 3′-UTR activities of FZD4 was decreased by miR-136-5p mimics, but had no effect in the same way on the activities of the FZD4 mutant 3′-UTR, which indicated the inhibition of the Wnt/β-catenin pathway. Therefore, our results suggested that the NLGN1-AS1/miR-136-5p axis could activate the classical Wnt/β-catenin signaling pathway by specifically suppressing FZD4.

## CONCLUSION

To sum up, we reveal for the first time the function of NLGN1-AS1 as a proliferative lncRNA in ccRCC both *in vitro* and *in vivo*. This study clarified the underlying mechanism of NLGN1-AS1 induction through inactivating FZD4/Wnt/β-catenin pathway. The identification and validation of our study offered new insights into investigation and clinical management of ccRCC. Therefore, this novel identified lncRNA NLGN1-AS1 might serve as a prognostic biomarker and potential therapeutic target for ccRCC treatment.
